# Macrolide resistance due to *erm*(55)

**DOI:** 10.1128/spectrum.02397-24

**Published:** 2025-01-16

**Authors:** David C. Alexander, Tayah Farquhar, Joshua M. E. Adams, Danae M. Suchan, Sean D. Workman, Richard J. Wallace Jr., Barbara A. Brown-Elliott, Omar M. El-Halfawy, Andrew D. S. Cameron

**Affiliations:** 1 Department of Medical Microbiology and Infectious Diseases, Max Rady College of Medicine, University of Manitoba, Winnipeg, Manitoba, Canada; 2 Cadham Provincial Laboratory, Diagnostic Services, Shared Health, Winnipeg, Manitoba, Canada; 3 Institute for Microbial Systems and Society, Faculty of Science, University of Regina, Regina, Saskatchewan, Canada; 4 Department of Biology, Faculty of Science, University of Regina, Regina, Saskatchewan, Canada; 5 Department of Chemistry and Biochemistry, Faculty of Science, University of Regina, Regina, Saskatchewan, Canada; 6 Mycobacteria/Nocardia Laboratory, The University of Texas at Tyler Health Science Center, UT Tyler School of Medicine, Tyler, Texas, USA; 7 Department of Microbiology and Immunology, Faculty of Pharmacy, Alexandria University, Alexandria, Egypt; MultiCare Health System, Tacoma, Washington, USA

**Keywords:** antibiotic resistance, *Mycobacterium*, molecular biology

## Abstract

Antimicrobial resistance (AMR) is a global threat. The identification and characterization of novel resistance genes is integral to AMR surveillance. The *erm*(55) gene was originally identified through whole genome sequencing of macrolide-resistant strains of *Mycobacterium chelonae*. The gene was annotated as a ribosomal methyltransferase, but its role as a determinant of macrolide resistance was not formally demonstrated. Three *erm*(55) alleles have now been documented. The plasmid-borne *erm*(55)^P^, the transposon-associated *erm*(55)^T^, and the chromosomal encoded *erm*(55)^C^ exhibit ≈82% amino acid sequence identity. Here, we confirm that, when expressed from plasmids in a macrolide-susceptible strain of *Escherichia coli*, all three *erm*(55) variants confer resistance to azithromycin and clarithromycin.

**IMPORTANCE:**

Macrolide antibiotics are often the only oral treatment option for infections with rapidly growing mycobacteria such as *Mycobacterium abscessus* and *Mycobacterium chelonae*. We previously identified three variants of a newly predicted macrolide resistance gene, *erm*(55), in *M. chelonae*, including the first case of a plasmid-mediated macrolide resistance in mycobacteria. The present study provides experimental evidence that the three *erm*(55) variants confer macrolide resistance and that each variant is unique in the degree to which it reduces susceptibility to clinically relevant macrolides.

## INTRODUCTION

Macrolides are broad spectrum antibiotics used to treat diverse bacterial infections. They inhibit bacterial protein synthesis by binding to the 50S ribosome (1). Macrolides were first isolated in the 1950s and erythromycin has been in use since 1952. Azithromycin and clarithromycin, second-generation macrolides derived from erythromycin, were developed in the 1980s and have been widely available since the 1990s (1).

Macrolides are a cornerstone of treatment for infections due to nontuberculous mycobacteria (NTM) (2). For species of rapidly growing mycobacteria (RGM), such as *Mycobacterium abscessus* and *Mycobacterium chelonae*, macrolides can be the only oral treatment option. Consequently, macrolide resistance is associated with clinical failure. Koh et al. (3) found that the cure (i.e., sputum conversion) rate for lung infections was 88% with macrolide-susceptible *M. abscessus*, but only 25% with clarithromycin-resistant *M. abscessus* strains (3).

Antimicrobial resistance to macrolides can occur via multiple mechanisms, including drug inactivation, antibiotic efflux, and changes to the bacterial ribosome (4). Mutations in the 23S rRNA or ribosomal protein genes can confer constitutive resistance. Inducible resistance is associated with methyltransferase enzymes encoded by erythromycin ribosome methylation (*erm*) genes. More than 50 classes of *erm* genes have been described. A standardized nomenclature for *erm* genes was established in 1999 (5). This scheme is based on amino acid identity such that sequences with ≥80% identity are given the same name, whereas those with ≤79% identity are assigned to different classes.

Functional chromosomal *erm* genes have been identified in multiple species of mycobacteria, including *erm*(37) in *Mycobacterium tuberculosis*, *erm*(38) in *Mycobacterium smegmatis*, *erm*(39) in the *Mycobacterium fortuitum* complex, *erm*(40) in both *Mycobacterium mageritense* and *Mycobacterium wolinskyi*, and *erm*(41) in *M. abscessus* subsp. *abscessus* and subsp. *bolletii* (6, 7). Recently, whole genome analysis of strains of *M. chelonae* with inducible clarithromycin resistance led to the discovery of *erm*(55) (8). Three alleles were documented: the plasmid-borne *erm*(55)^P^, the transposon-associated *erm*(55)^T^, and the chromosomal-encoded *erm*(55)^C^. Because the three variants exhibit ≈82% amino acid sequence identity, they share the same *erm*(55) designation.

Although annotated as a rRNA methyltransferase and associated with clarithromycin-resistant strains of *M. chelonae*, the role of *erm*(55) as a determinant of macrolide resistance has not been formally evaluated. Here, we test whether *erm*(55) confers macrolide resistance.

## RESULTS

The coding regions for each *erm*(55) variant were cloned into the low-copy number plasmid pWSK2 under transcriptional control of the *lacZ* promoter. The plasmids were then transformed and expressed in a strain of *E. coli* M6394 with the deletion of nine TolC-dependent efflux pumps (herein denoted M6394Δ9) (9–11). Most *E. coli* strains, including derivatives of *E. coli* MG1655 commonly used in laboratories, are intrinsically resistant to macrolides. The outer membrane barrier effect combined with the action of multiple efflux pumps results in poor penetration and low intracellular accumulation of these drugs. The deletion of nine TolC-dependent efflux pumps increases the permeability of the outer membrane and reduces active efflux such that *E. coli* M6394Δ9 is macrolide susceptible. The minimum inhibitory concentration (MIC) of the macrolide azithromycin decreases from 4 µg/mL for *E. coli* MG1655 to 0.5 µg/mL for *E. coli* M6394Δ9.

Broth microdilution was used to determine the MIC for clarithromycin and azithromycin. Antibiotic susceptibility testing (AST) results are summarized in Table 1 and Fig. S1. The control plasmid vector pWSK29 had no impact on the macrolide susceptibility of *E. coli* M6394Δ9. Conversely, the presence of any of the cloned *erm*(55) genes increased the MIC for both clarithromycin and azithromycin. The increased resistance was more pronounced against clarithromycin, with MIC shifts of 16-fold in the presence of *erm*(55)^P^ and 256-fold with both *erm*(55)^C^ and *erm*(55)^T^. In comparison, the MIC shifts for azithromycin were 4-, 8-, and 32-fold in the presence of *erm*(55)^P^, *erm*(55)^C^, and *erm*(55)*^T^*, respectively.

**TABLE 1:** Antibiotic susceptibility testing results for clarithromycin and azithromycin

*E. coli* M6394Δ9	MIC (µg/mL)	
containing	Clarithromycin	Azithromycin
No plasmid	2	0.5
pWSK29	2	0.5
pWSK29::*erm*(55)^P^	32	2
pWSK29::*erm*(55)^C^	512	4
pWSK29::*erm*(55)^T^	512	16

## DISCUSSION

Macrolide resistance in RGM species is typically attributed to chromosomal *erm* genes and/or mutations in the 23S rRNA gene. Historically, *M. chelonae* was not known to contain *erm* genes, but strains exhibiting inducible clarithromycin resistance and wild-type 23S rRNA loci were recently recognized (8). Systematic whole genome sequencing of these isolates led to the discovery of a putative *erm* gene (8). Because this novel sequence exhibited <65% amino acid identity to previously described sequences, it was assigned a new designation: *erm*(55). Three variants were documented: the plasmid-borne *erm*(55)^P^, the transposon-associated *erm*(55)^T^, and the chromosomal-encoded *erm*(55)^C^. The variants have distinct gene and promoter sequences but are similar enough (≈82% amino acid identity) to share the same *erm*(55) classification.

In the current study, the coding regions for each *erm*(55) variant were cloned into low copy number plasmid pWSK2 and expressed in the macrolide susceptible strain, *E. coli* M6394Δ9 (9–11). As indicated in Table 1, the MICs for clarithromycin and azithromycin for *E. coli* M6394 Δ9 are 2 and 0.5 µg/mL, respectively. The control pWSK29 vector had no impact on these MICs. However, the cloned *erm*(55) genes increased the MICs for both clarithromycin and azithromycin. Notably, the different *erm*(55) sequences conferred different antibiotic susceptibility profiles such that the MIC for clarithromycin was 512 µg/mL for both *erm*(55)^T^ and *erm*(55)^C^ but only 32 µg/mL for *erm*(55)^P^. With azithromycin, the trend was similar such that *erm*(55)^T^ was associated with the highest MIC (16 µg/mL) and *erm*(55)^P^ the lowest (2 µg/mL), but the *erm*(55)^C^ result was intermediate (4 µg/mL).

These MIC results clearly demonstrate that, consistent with its predicted function, *erm*(55) is associated with changes in macrolide susceptibility. However, there are several limitations to this work. We did not formally demonstrate that *erm*(55) expression changes the methylation state of the *E. coli* ribosome. Although the variant-specific differences in MIC are reproducible, additional genetic and biochemical experiments are required to understand why they occur. Although we have not yet attempted these experiments in any mycobacterial species, previous work indicates that the *erm*(55)^T^ and *erm*(55)^C^ alleles also confer higher levels of clarithromycin resistance in *M. chelonae*.

In Table 1 of the original study by Brown-Elliott et al. (8), strains containing the *erm*(55)^P^ plasmid had 3-day clarithromycin MICs ≤ 4 µg/mL (8). Only after extended incubation were the clarithromycin MICs ≥ 16 µg/mL. In contrast, *M. chelonae* strains encoding *erm*(55)^T^ or *erm*(55)^C^ had 3-day clarithromycin MICs ≥ 16 µg/mL. In *M. chelonae*, allele-specific differences in the *erm*(55) promoter region may influence gene expression and inducible macrolide resistance. Yet, in the *E. coli* system, the genes are expressed from the same promoter, which suggests coding sequence differences may also influence protein function. Structural studies of Erm38 from *M. smegmatis* indicate that arginine residues in the C-terminal region of the protein play a key role in binding the rRNA substrate (12). In the C-terminal region of Erm55, there is an Arg206→Ser substitution, with Erm55^T^ and Erm55^C^ sharing Arg206 and Erm55^P^ containing Ser206. Proper structural studies are required, but Ser206 in Erm55^P^ may result in weaker binding to the rRNA substrate and, thus, slower or less efficient ribosomal methylation.

Using a genomics-based approach, we previously identified DNA sequences consistent with a novel *erm* gene (8). Here, we have provided experimental confirmation that *erm*(55) is a determinant of macrolide resistance. Macrolide resistance compromises the treatment of NTM infections. A chromosomal *erm* gene may confer resistance to a particular bacterial strain, but plasmids and transposons are mobile elements with the potential to spread *erm* genes between species. Historically, plasmids were not thought to contribute to antibiotic resistance in mycobacteria. However, in the past decade, two resistance plasmids have been described: pMAB01, which confers aminoglycoside resistance in *M. abscessus* (13), and now, the *erm*(55) plasmid that confers macrolide resistance in RGM species, including *M. chelonae* (8).

## MATERIALS AND METHODS

### Construction of *erm*(55) plasmids

Low-copy plasmids containing each *erm*(55) gene were constructed with the New England Biolabs Hifi DNA Assembly Protocol (NEB, MA, USA). Gene sequences were based on *erm*(55)^P^ from *M. chelonae* MCHL-2067831 (OQ656455.1), *erm*(55)^T^ from *M. chelonae* MCHL-2173373 (OQ656456.1), and *erm*(55)^C^ from *M. chelonae* MCHL-2170968 (OQ656457.1). Oligonucleotides for the amplification of the pWSK29 backbone and three *erm*(55) alleles were designed using the NEBuilder Assembly tool and synthesized by IDT. Primer sequences are listed in Tables 2 and 3. PCR was performed in 50 µL reactions using 25 µL Q5 High-Fidelity DNA Polymerase Master Mix (NEB, MA, USA), 5 µL (10 µM) primers, and 4 µL (≈100–200 ng/µL) template DNA. PCR products were visualized by agarose gel electrophoresis and amplicons of appropriate size were extracted using the Monarch DNA Gel Extraction kit (NEB, MA, USA). The purified amplicons were quantified using the Qubit dsDNA BR Assay kit (Invitrogen, MA, USA).

**TABLE 2:** Primer sequences for cloning of *erm*(55) genes

Name	Amplicon size (bp)	Direction	Primer sequence (5′-3′)
*erm*(55)^P^	833	Forward	ggaaacagctatgcctactttccgtgctg
		Reverse	aaccatcacctcatcgcccgttcctcgaac
*erm*(55)^C^	833	Forward	ggaaacagctatgcctacttttcacgctg
		Reverse	aaccatcacctcatcgccaattcctcgaac
*erm*(55)^T^	833	Forward	ggaaacagctatgcctacttatcgtgccg
		Reverse	aaccatcacctcatcgccgactcctcgaac

**TABLE 3:** Primer sequences for pWSK29 backbone

Name	Amplicon size (bp)	Direction	Primer sequence (5′-3′)
pWSK29–*erm*(55)^P^	4920	Forward	cgggcgatgaggtgatggttcacgtagtg
		Reverse	aagtaggcatagctgtttcctgtgtgaaattg
pWSK29–*erm*(55)^C^	4920	Forward	ttggcgatgaggtgatggttcacgtagtg
		Reverse	aagtaggcatagctgtttcctgtgtgaaattg
pWSK29–*erm*(55)^T^	4920	Forward	tcggcgatgaggtgatggttcacgtagtg
		Reverse	aagtaggcatagctgtttcctgtgtgaaattg

NEB Hifi DNA Assembly Protocol enabled cloning of each *erm*(55) gene variant into the pWSK29 backbone. Plasmid DNA was transformed into *E. coli* NEB5α following the manufacturer’s high-efficiency protocol. Transformants were selected on LB agar containing ampicillin (100 µg/mL). For each construct, a single colony was picked and cultured in LB broth and then tested by colony PCR with the same allele-specific *erm*(55) primers used for the initial cloning. The identity of each construct was also confirmed by DNA sequencing (Plasmidsaurus Inc, CA, USA).

### Transformation of *E. coli* M6394Δ9 with pWSK29

AST experiments were performed in *E. coli* M6394Δ9 (10). Due to deletions in nine TolC-dependent efflux pump systems (Δ*acrB* Δ*acrD* Δ*acrEF::spc* Δ*emrB* Δ*emrY* Δ*entS::cam* Δ*macB* Δ*mdtC* Δ*mdtF*), this *E. coli* strain is macrolide susceptible.

Competent cells were prepared by culturing *E. coli* M6394Δ9 in LB broth overnight at 37°C with shaking at 200 rpm. Cells were harvested in the mid-log phase (OD_600_ 0.4–0.6) and then chilled on ice for 10 min. Cells were pelleted by centrifugation for 10 min at 5000 rpm at 4°C, the supernatant was discarded, and the cells were resuspended in 1 mL of 0.1 M CaCl_2_. Once resuspended, an additional 25 mL of CaCl_2_ was added, cells were chilled on ice for 1 h, and then centrifuged at 4°C for 10 min at 5000 rpm. The supernatant was discarded and the cells were resuspended in 1.5 mL of 0.1 M CaCl_2_ and 15% glycerol. Competent cells were divided into 70 µL aliquots, immediately frozen in liquid nitrogen, and stored at –80°C.

For transformation, competent cells were thawed on ice and 1 µL of recombinant plasmid DNA was added to each aliquot. They were incubated on ice for 30 min, heat-shocked at 42°C for 30 s, and then chilled on ice for 2 min. Next, 1 mL of Super Optimal broth with Catabolite repression (SOC) media was added and the culture was incubated for 1 h at 30°C. Finally, cells were plated onto LB agar containing ampicillin.

### Antibiotic susceptibility testing (AST)

AST was performed by microdilution in Mueller Hinton Broth (14). MIC assays for azithromycin (Sigma-Aldrich) and clarithromycin (TCI America, OR, USA) were conducted in 96-well polystyrene plates with a final volume of 100 µL/well. Inoculum density was determined using a spectrophotometer (Thermo Scientific Spectronic 200) and adjusted to an optical density at 600 nm (OD_600_) of 0.001 (≈5 × 10^5^ colony forming units per mL). Cultures were incubated at 37°C with shaking at 600 rpm. Growth of the bacterial cultures was determined at 24 h using OD_600_ measurements. For each plasmid construct, the final MIC result was based on three independent AST experiments, each performed in triplicate (i.e., nine measurements).

## AFTER EPUB

*[This article was published on 16 January 2025 with an error in the Results section and several in the Author Contributions. The Results section and Author Contributions were corrected in the current version, posted on 29 January 2025.]*

## SUPPLEMENTAL MATERIAL

Fig. S1 spectrum.02397-24-s0001.pdf Antibiotic susceptibility testing for clarithromycin and azithromycin.

ASM does not own the copyrights to Supplemental Material that may be linked to, or accessed through, an article. The authors have granted ASM a non-exclusive, world-wide license to publish the Supplemental Material files. Please contact the corresponding author directly for reuse.
