# Supplementary material for: Macrolide resistance due to erm(55)
Source: Microbiol Spectr. 2025 Jan 16;13(3):e02397-24. doi: 10.1128/spectrum.02397-24 (PMC11878018; doi:10.1128/spectrum.02397-24)
Supplement: Fig. S1 — Antibiotic susceptibility testing for clarithromycin and azithromycin. [file spectrum.02397-24-s0001.pdf]

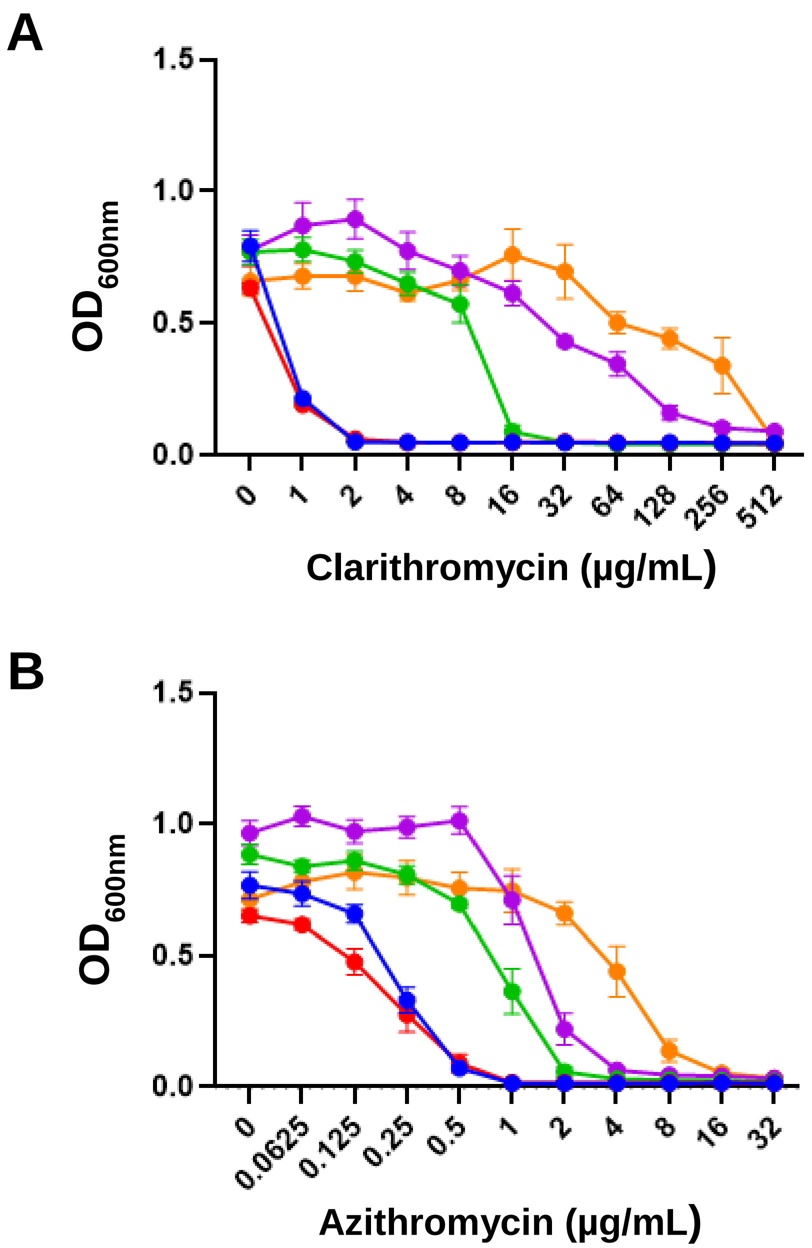

Figure S1: Antibiotic susceptibility testing for **A) Clarithromycin** and **B) Azithromycin.** MICs were determined by broth microdilution for five strains, including: *E. coli* M6394Δ9 (no plasmid), *E. coli* M6394Δ9 pWSK29, *E. coli* M6394Δ9 pWSK29::*erm*(55)^{P}, *E. coli* M6394Δ9 pWSK29::*erm*(55)^{C} and *E. coli* M6394Δ9 pWSK29::*erm*(55)^{T}. Growth of the bacterial cultures was determined at 24 h using OD_{600} measurements. The final MIC result was based on three independent AST experiments, each performed in triplicate. Results from one representative set of experiments are shown.
